# Adsorption Efficiency of Cadmium (II) by Different Alkali-Activated Materials

**DOI:** 10.3390/gels10050317

**Published:** 2024-05-05

**Authors:** Nataša Mladenović Nikolić, Ljiljana Kljajević, Snežana S. Nenadović, Jelena Potočnik, Sanja Knežević, Sabina Dolenec, Katarina Trivunac

**Affiliations:** 1 Department for Materials, “Vinča“ Institute of Nuclear Sciences—National Institute of the Republic of Serbia, University of Belgrade, 11 000 Belgrade, Serbia; ljiljana@vin.bg.ac.rs (L.K.); msneza@vin.bg.ac.rs (S.S.N.); sanja.knezevic@vin.bg.ac.rs (S.K.); 2 Department of Atomic Physics, “Vinča“ Institute of Nuclear Sciences—National Institute of the Republic of Serbia, University of Belgrade, 11 000 Belgrade, Serbia; jpotocnik@vin.bg.ac.rs; 3 Slovenian National Building and Civil Engineering Institute, Dimičeva ulica 12, 1000 Ljubljana, Slovenia; sabina.dolenec@zag.si; 4 Department of Geology, Faculty of Natural Sciences and Engineering, University of Ljubljana, Aškerčeva ulica 12, 1000 Ljubljana, Slovenia; 5 Department of Analytical Chemistry and Quality Control, Faculty of Technology and Metallurgy, University of Belgrade, 11 000 Belgrade, Serbia; trivunac@tmf.bg.ac.rs

**Keywords:** fly ash, metakaolin, wood ash, amorphous gel, adsorption kinetics, cadmium (II)

## Abstract

The objective of this study was to demonstrate the potential utilization of fly ash (FA), wood ash (WA), and metakaolin (MK) in developing new alkali-activated materials (AAMs) for the removal of cadmium ions from waste water. The synthesis of AAMs involved the dissolution of solid precursors, FA, WA, and MK, by a liquid activator (Na_2_SiO_3_ and NaOH). In concentrated solutions of the activator, the formation of an aluminosilicate gel structure occurred. DRIFT spectroscopy of the AAMs indicated main vibration bands between 1036 cm^−1^ and 996 cm^−1^, corresponding to Si-O-Si/Si-O-Al bands. Shifting vibration bands were seen at 1028 cm^−1^ to 1021 cm^−1^, indicating that the Si-O-Si/Si-O-Al bond is elongating, and the bond angle is decreasing. Based on the X-ray diffraction results, alkali-activated samples consist of an amorphous phase and residual mineral phases. The characteristic “hump” of an amorphous phase in the range from 20 to 40° 2*θ* was observed in FA and in all AWAFA samples. By the XRD patterns of the AAMs obtained by the activation of a solid three-component system, a new crystalline phase, gehlenite, was identified. The efficiency of AAMs in removing cadmium ions from aqueous solutions was tested under various conditions. The highest values of adsorption capacity, 64.76 mg/g (AWAFA_6_), 67.02 mg/g (AWAFAMK_6_), and 72.84 mg/g mg/g (AWAMK_6_), were obtained for materials activated with a 6 M NaOH solution in the alkali activator. The Langmuir adsorption isotherm and pseudo-second kinetic order provided the best fit for all investigated AAMs.

## 1. Introduction

In the present day, many industrial processes, including pesticide manufacturing, petroleum refining, electronic industry activities, paint, leather pigment and rechargeable battery production, and mining, contribute significantly to the discharge of heavy metals into rivers, leading to considerable environmental damage [1,2,3]. Cadmium, like other toxic heavy metals (arsenic (As), copper (Cu), mercury (Hg), nickel (Ni), lead (Pb), and chromium (Cr)) is identified as posing serious threats to living organisms [4,5]. These heavy metals are non-biodegradable, tend to accumulate in living tissues upon entering the human body, and pose serious health risks, particularly to the kidneys, pulmonary, cardiovascular, and musculoskeletal systems [6]. High Cd concentrations in some drainage channel sediments in Vojvodina (Serbia) near highly productive agricultural land areas were determined by Savić et al. (2015) [7]. According to the research conducted by Nikolić et al. in 2020, the enrichment of cadmium was observed in six reservoirs in Serbia (Garaši, Vlasina, Perućac, Zaovine, Međuvršje, and Sava Lake) [8]. Also, Sakan et al. in 2011 observed the highest content of cadmium in the Ibar, Tisa, Sava, South Morava, Nišava, Kolubara, and Pek river sediments [9]. Therefore, the decontamination of heavy metals from aqueous solutions is crucial for safeguarding public health and the environment. Several techniques have been employed to remove heavy metal ions from wastewater [10,11,12,13]. Among these, adsorption stands out as an effective physicochemical method due to its ease of handling, low capital cost, high efficiency, and suitability for both batch and continuous processes [14,15].

Various materials, including biochar [10], agricultural waste biomass [16,17], bentonite clay [18,19], fly ash [20,21], and red mud [22] are investigated as efficient, low-cost, and eco-friendly adsorbents for removing cadmium ions from wastewater. Alkali-activated materials, as relatively new materials, have gained significant attention for their excellent immobilization effect and high capacity for cation exchange, thus they can be used as adsorbents [23,24,25]. These materials exhibit eco-friendly properties and high efficiency in removing various toxic metals and dyes due to their porous, amorphous, and heterogeneous microstructure. AAMs demonstrate good surface properties for the adsorption process and possess favorable mechanical, thermal, and chemical resistance [26,27,28,29,30].

AAMs are synthesized under highly alkaline conditions from aluminosilicate or calcium aluminosilicate precursors, as well as metakaolin, coal fly ash [20,31,32,33], ground granulated blast furnace slag [34], rice husk ash, and palm oil fuel ash [35,36]. Also, a certain amount of these precursors can be replaced by ash from the burning of plant mass, e.g., wood [18,31,37,38]. The alkali activation process occurs on the amorphous phase of the precursor. When solid aluminosilicate precursor particles come into contact with the alkali activator solution, they are dissolved by the alkaline hydrolysis reaction. The time for the supersaturated aluminosilicate solution to form a continuous gel varies considerably with the type of raw materials, composition of the alkali-activated solution, and process conditions [39]. Following gelation, the system undergoes further rearrangement and reorganization. As the gel network’s connectivity enhances, it leads to the formation of the three-dimensional aluminosilicate network. The result of the activation reactions essentially consists of two consecutive and controlling processes or stages in the entire process of alkaline activation. These are nucleation, or the dissolution of aluminosilicate material, and the formation of polymeric species, namely polymeric three-dimensional gel networks [40].

All around the world, power plants produce more than 500 million tons of fly ash per year; out of that, around 30% is reused [41]. In Serbia, thermal power plants every year produce around 7 million tons of fly ash and slag, but only 3% is used in the cement industry [42]. Additionally, individual household fireplaces produce a significant amount of ash, which is disposed of in environment and can cause a serious ecological and health problems. The quantity, quality, and environmental hazards of biomass ash are influenced by the characteristics of the biomass, combustion process, and operation conditions [37]. Fly and wood ash themselves are pollutants of the water system and also for the soil and air. Their use as a recyclable material is of immeasurable importance from an environmental standpoint. Also, the utilization of combustion waste or byproducts such as fly and wood ash as precursors for AAMs production offers environmental benefits, including reduced carbon dioxide emissions, minimizing the use of natural resources, providing alternatives to natural minerals, and the responsible disposal of ashes [36,37,38]. The ashes have pozzolanic properties (presence of CaO) and exhibit the durability of aluminum–silicon structures in a wide temperature range (up to 900 °C), which can be attributed to non-agricultural applications of ash. The ability to adsorb cations, high cation-exchange capacity, and high mobility of some metals (Pb, Cr, Co) may effect is a possible application of wood ash for the synthesis of alkali-activated materials [43]. Metakaolin (MK) is a commonly used aluminosilicate material for the synthesis of geopolymer/AAMs-based adsorbents because it has unique adsorption properties [44]. However, MK possesses characteristics that limit its use, such as weak rheological properties, processing, increased cost, high water demand, quick hydration reactions, and high heat gain in the initial phases [45,46,47]. This is one of the reasons for replacing MK with some other aluminosilicate material, such as fly ash. The presence of metal oxides in the industrial waste (fly ash) increases the active sites on the surface of the AAMs and offers a contribution of cation exchange, ion precipitation, and ion complexation with heavy metal [46,48,49]. The addition of wood ash, given that it is rich in calcium and carbon, has the role of stabilizing the aluminosilicate matrix in a short period of time.

The objective of this study was to explore an environmentally friendly approach by reusing fly and wood ash in combination with calcined clay-metakaolin to create novel materials capable of efficiently removing cadmium from wastewater. The emphasis is on developing a useful new product with minimal raw material, energy, and human resource consumption, while also minimizing the negative impact on the environment. In addition, this research aims to highlight the potential of utilizing waste materials such as fly ash, which pose a major environmental problem, both for the residents of places where tons of this waste are directly disposed of, but also for the wider community, which should be aware that the problem of waste disposal is such that tomorrow those who did not suffer the consequences today will find themselves in a similar situation.

## 2. Results and Discussion

Before investigating the removal efficiency of the applied adsorbents, structural, mineralogical, and microstructural analyses were performed.

### 2.1. Structural, Mineralogical, and Microstructural Analyses

#### 2.1.1. DRIFT Analysis

**Figure 1:**
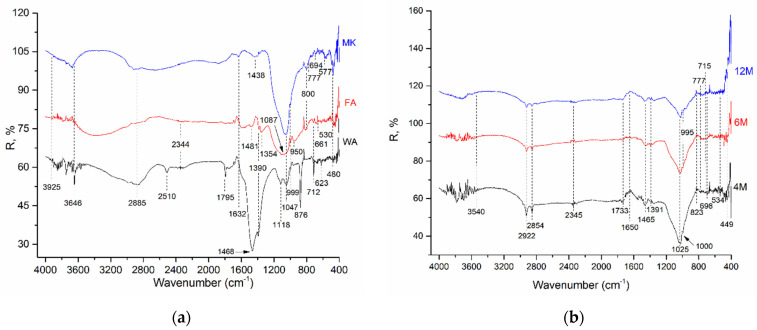

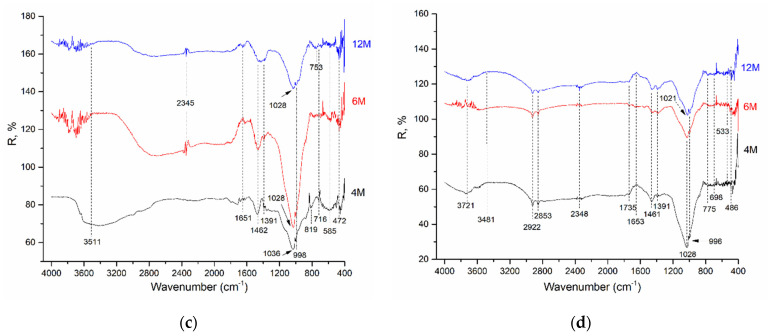
DRIFT spectrum of the investigated samples: (**a**) WA, FA, and MK; (**b**) AWAFA; (**c**) AWAMK; and (**d**) AWAFAMK.

DRIFT spectra of all investigated samples are presented on the Figure 1. Figure 1a describes the DRIFT spectra of WA, FA, and MK, while Figure 1b–d show the DRIFT spectra of different AAMs.

The bands in the range of 3920–3400 cm^−1^ are attributed to O-H and H-O-H stretching vibrations. The bands at 1650 cm^−1^ correspond to H-O-H bending vibrations from the adsorbed water molecule [50,51,52,53]. The acute band (~3800 cm^−1^) was derived from the regularly distributed group OH in the structure of WA. The strong band ~3600 cm^−1^ is related to a randomly placed hydroxyl group in the structure. The peaks at 1468 cm^−1^, 876 cm^−1^, and 712 cm^−1^ in the WA spectrum were attributed to the bending and asymmetric stretching vibrations of carbonate presented in raw WA [54], while the band around 1510 cm^−1^ could be related to the C=C vibration which is characteristic of lignin that is most often obtained from wood [55]. The presence of peaks related to the vibrations of aluminosilicate and silicate structures are: Si-O (1118 cm^−1^, 1047 cm^−1^), symmetric bridge stretching vibration, Al-Si-O (623 cm^−1^) symmetric stretching vibration, as well as O-Si-O bending vibrations related to the presence of silica glass and quartz (480 cm^−1^). The band area ~3500 and ~1600 cm^−1^ for FA samples indicates the (-OH) bond stretching and the (H-O-H) vibration bending of bound water molecules while they are entrapped in polymeric framework cavities and absorbed on the surface. The bands around ~1000 cm^−1^ indicate the gains in the Si-O-Si bands typical of quartz, and the 800–500 cm^−1^ band indicates the symmetric stretching of the Si-O-Si and Al-O-Si bonds which describe the formation of an amorphous gel to semi-crystalline aluminosilicate materials. The bands at 800 cm^−1^ in FA refer to external ring vibrations formed when tetrahedral AlO_4_ and SiO_4_ are linked by oxygen atoms [50,56], while the band observed at 530 cm^−1^ represents mullite [57]. The bands below 500 cm^−1^ indicate the bending vibrations of the Si-O-Si and O-Si-O bonds (<500 cm^−1^) of the FA structure. These results confirm the chemical composition of FA using XRF analysis.

The DRIFT spectra of MK show the most characteristic peaks observed at 1042 cm^−1^, concerning the band attributed to the asymmetric stretching vibrations of Si–O–Si and Al–O–Si. The bands at 800 cm^−1^, 777 cm^−1^, and 694 cm^−1^ present in the DRIFT spectra of MK, related to the stretching vibration of 6-fold coordinated Al (VI)-OH and 6-fold coordinated Al(VI)-O, disappeared after polymerization.

Further, AAMs formed from mixtures of solids are characterized WA/FA and WA/MK, as well as the three-component mixture WA/FA/MK. The characteristic vibrations for newly formed AAMs occur at other wavenumbers. It must be taken into account that each component makes a certain contribution, so the analysis is very demanding and complex.

In all spectra of AAMs, characteristic reflection bends were detected. At a wavenumber of ~3400 cm^−1^–3500 cm^−1^, it can be noticed that the bends corresponded from the stretching vibration of the -OH group. Also, the bends originated from the stretching vibrations of the -CH_2_ and -CH_3_ functional groups at ~2900 cm^−1^, and bending vibrations from the absorbed water of the -OH group at ~1650 cm^−1^ were present. The broad band at about 1000 cm^−1^ represents the most important peak in the spectra of the AAMs [58,59]. This band originates from the Si-O-X asymmetric vibration, where X represents Al or Si in tetrahedral coordination. In the DRIFT spectra of FA-based AAMs, a broad band at about 1000 cm^−1^ originates from the collective signal of various silicate and aluminosilicate components present in the material: quartz, mullite, and unreacted aluminosilicate glass phase from the FA and aluminosilicate gel geopolymer [60]. In the case of amorphous SiO_2_ (X = Si), this band is located at about 1100 cm^−1^. The shift of this vibrational maximum to lower wavenumbers indicates the lengthening of the Si-O-X bond or the reduction in the bond angle. During the alkaline activation process of FA, the position of the vibrational maximum originating from Si-O-X asymmetric vibrations shifts to lower values of the wavenumber (Figure 1b,c), which indicates that there is a dissolution of FA in the alkaline activator, an increase in the concentration of non-binding oxygen ions, and the inclusion of Al into a silicate structure [61]. The shift in the position of the vibrational maximum of the S-O-X asymmetric vibration in the spectrum of the AAMs indicates changes in the relative Si/Al ratio in a gel phase of the AAMs [62]. After alkaline activation of raw materials, the band shift from 1047 cm^−1^ to wavenumbers between 1021 cm^−1^ and 1036 cm^−1^ can be attributed to the asymmetric stretching vibrations of Si-O-Si/Si-O-Al, which are characteristic of AAMs [63,64]. Carbonate stretches appeared at approximately 1462–1468 cm^−1^ and 1390 cm^−1^. The assumption is the formation of sodium carbonate due to the action of NaOH as part of the alkali activator and residual calcium carbonate (CaCO_3_). Also, it could be formed after a reaction with carbon dioxide from the air [51,53,54,64,65]. The band ranging from 1100 to 900 cm^−1^ is attributed to the stretching of Si-O-Si/Si-O-Al bonds [50]. A weak band at 849–878 cm^−1^ was observed in all AAMs. It was assigned to the bending vibration of Si–OH. The presence of Si-OH in the AAMs will cause a decrease in the degree of polycondensation reaction, and thus a reduction in the mechanical strength of the AAMs [66]. In the AAMs, the bands around the 450 cm^−1^ regions are associated with the internal deformation vibration of the Si-O/Al-O bonds, also known as the in-plane bending mode [55]. The bending vibrations of Si-O-Al/Si-O-Si between 450 cm^−1^ and 480 cm^−1^ in raw and AAMs materials were also observed [50,51,52,53,54,55,56,57,58,59,60,61,62,63,67]. In the AAMs, which consist of FA, it can be seen that the bands around 2922 cm^−1^ and 2855 cm^−1^ are related to asymmetric and symmetric methyl and methylene stretching groups [2,55]. Also, small bands at 2350–2345 cm^−1^ in the raw materials WA and FA, and AAMs which consist of those raw materials, are probably due to the infrared band position of HCO_3_^-^ ions [56,68,69]. The impact of a different concentration of the activation solution between the same AAMs was noticeable (Figure 1b–d). For example, in AWAFA_4_ the bands located at 1000 cm^−1^ and 449 cm^−1^ correspond to SiO_4_/AlO_4_ tetrahedral asymmetric stretch vibrations and the bending vibrations of Si-O- groups on the surface, respectively, while for AWAFA_6_ and AWAFA_12_ samples the tetrahedral asymmetric stretch vibrations of SiO_4_/AlO_4_ were shifted to 995 cm^−1^ [2]. Additionally, it is observed that the main band for AWAMK_4_ is located at 1036 cm^−1^ and shifted to 1028 cm^−1^ for AWAMK_6_ and AWAMK_12_. The vibration band at 1028 cm^−1^ is observed in the spectra of the AAMs AWAFAMK_4_ and AWAFAMK_6_. This could be attributed to the asymmetric stretching vibrations of silica Si-O-Si. However, the AWAFAMK_12′_s main vibration band was shifted to 1021 cm^−1^, indicating that the Si-O-Si/Si-O-Al bond is elongating, and the bond angle is decreasing [63,64].

#### 2.1.2. XRD Analysis

**Figure 2:**
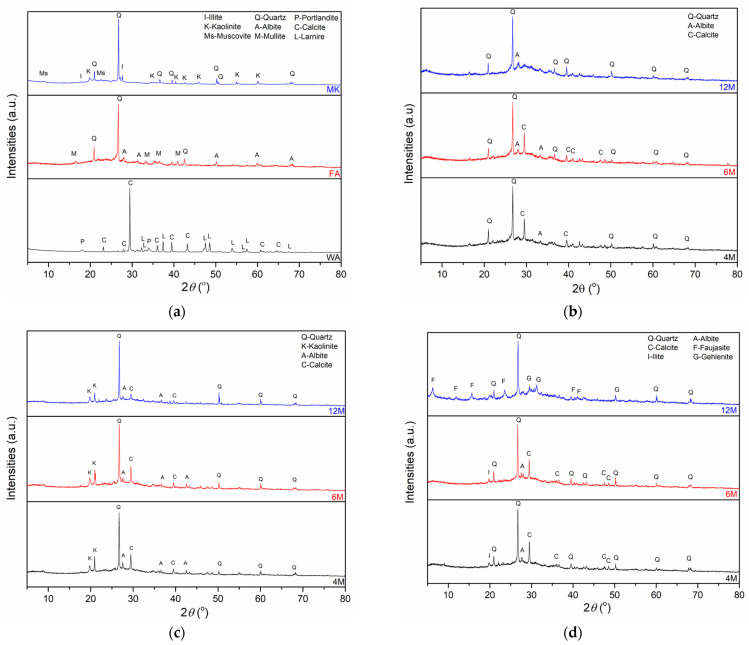
XRD pattern of (**a**) raw materials, and (**b**) AWAFA, (**c**) AWAMK, and (**d**) AWAFAMK.

The mineralogy composition of the investigated samples was determined by X-ray analysis. The X-ray diffraction patterns of the raw materials MK, FA, and WA and AAMs at 28 days are shown in Figure 2. Figure 2a shows the XRD pattern of WA, FA, and MK, while Figure 2b–d show the XRD patterns of AAMs.

The phase analysis outcomes given by XRD analysis of WA, FA, and MK contain different crystal phases. Quartz is a mineral that can be considered as primary, especially in cases where samples originated from fuels such as coal and lignite which contain high amounts of silicon dioxide. A diffraction peak at approximately 30° 2*θ* was noticed in WA, and it originated from calcite (PDF No: 01-071-3699). As shown by XRF analysis (Section 4.2.1), WA is rich in calcium. In association with calcite, larnite (PDF No. 01-083-0464) and portlandite (PDF No. 00-044-1481) occurred in WA. Portlandite manifests itself at low or medium combustion temperatures, by calcite heating [70], and only WA consisted of this mineral. The phase analysis of FA showed the presence of mullite (PDF No. 00-015-0776), quartz (PDF No: 00-033-1161), and albite (PDF No: 00-009-0466). In addition to quartz, illite (PDF No: 00-043-0685), kaolinite (PDF No. 01-072-5860), and muscovite (PDF No. 01-070-975) phases were determined in MK.

The XRD patterns of samples of AWAFA and AWAMK are displayed in Figure 2b,c. Based on the X-ray diffraction results, alkali-activated samples consist of an amorphous phase and residual mineral phases that did not fully decompose in the alkali activation process. Also, the characteristic “hump” [71] of an amorphous phase in the range from 20 to 40° 2*θ* is observed in FA and in all AWAFA samples. Almost all of the crystalline phases in these AAMs are the same as those in the precursors. Muscovite was not identified in AWAMK, although it is present in MK. The influence of the molarity of the alkaline activator on the appearance of new crystalline phases in AWAFA and AWAMK is not observed in comparison with the mineralogical composition of AWAFAMK samples (Figure 2d). Portlandite and larnite from WA, muscovite from MK, and mullite from FA are dissolved during alkali activation, and they were not identified in the determined AAMs.

Regarding the XRD patterns of AWAFAMK, XRD analysis identified new crystalline phases in the AWAFAMK_12_. The new crystalline phase in this sample is gehlenite (PDF: 01-087-0968) which originates from carbonate-silicate-spinel reactions between illite and calcite [72]:

KAl_2_(Si_3_Al)O_10_(OH)_2_ + 6 CaCO_3_ → 3 Ca_2_Al_2_SiO_7_ + 6 CO_2_ + 2 H_2_O + K_2_O + 3 SiO_2_    Illite        Calcite     Gehlenite

Based on Figure 2d, the presence of crystal phases of illite and calcite can be observed in AAMs obtained by the activation of FA, MK, and WA with a lower pH solution of alkaline activators. By applying an alkaline activator where the concentration of the NaOH solution is 12 M to the precursor mixture where there is an equal ratio of FA and MK with the addition of 10% WA, the formation of gehlenite, as a new crystalline phase, occurred, while illite and calcite phases were not identified in this sample.

#### 2.1.3. FESEM-EDS Analysis

**Figure 3:**
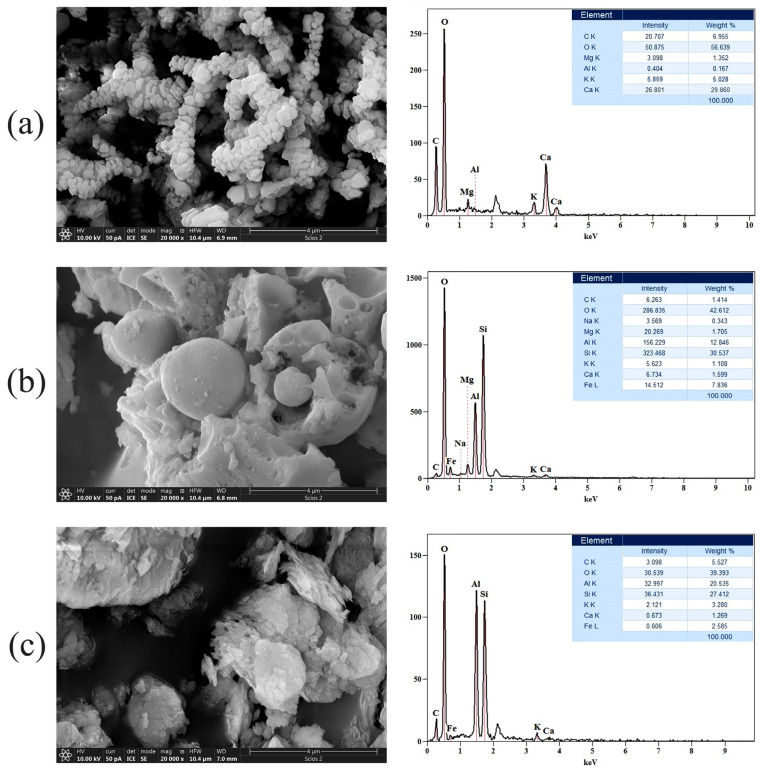
FESEM micrographs (**left** side) and corresponding EDS spectra (**right** side) of raw materials: (**a**) WA, (**b**) FA, and (**c**) MK; EDS analysis was performed on the entire surface depicted in Figure 3 (**left** side).

The surface morphologies of the raw materials WA, FA, and MK were studied using FESEM and the resulting micrographs are given in Figure 3 (left side). As illustrated in Figure 3a, WA consists of intertwined chains with irregularly shaped, but uniformly sized particles. WA grains are characterized by a complex morphology. The size of the individual grains is less than 1 μm. The finest grains occur in the form of aggregates, which are building larger particles. Grains have both angular and rounded edges. The interiors of larger particles are filled with smaller ash particles.

On the other hand, Figure 3b shows that FA is composed of characteristic spherical particles of varying sizes, surrounded by a shapeless matrix, whereas Figure 3c depicts the common morphology of the MK, indicating material with a dense matrix of differently shaped and flake-like particles. In order to analyze and check the chemical composition of the samples, EDS analysis was also performed and the acquired results are given in Figure 3 (right side). EDS spectra were collected in the energy range of 0.1–10 keV. The results revealed that the analyzed samples were mainly comprised of oxygen (O), aluminum (Al), silicon (Si), carbon (C), potassium (K), and sodium (Na). Furthermore, in the acquired spectra, some additional peaks were observed. For instance, a peak attributed to magnesium (Mg) occurs in the WA and FA samples and a sodium (Na) peak appears in the FA sample, while iron (Fe) peaks are detected in the FA and MK samples, and the relatively weak signal intensities are in magnesium, potassium, calcium, and iron. Particles of FA have more than 50% chemical proportions of (Al), (Si), and (O) with strong signal intensities. Some of these particles are called amorphous aluminosilicate spheres. The microstructure of the AAMs formed after 28 days of curing is investigated. Different types of morphologies formed during the processes of alkali activation (polymerizations/condensations/curing-hardening). Subsequently, the NaOH alkaline activator drains out the aluminosilicate particles, resulting in polymeric gel formation [24].

**Figure 4:**
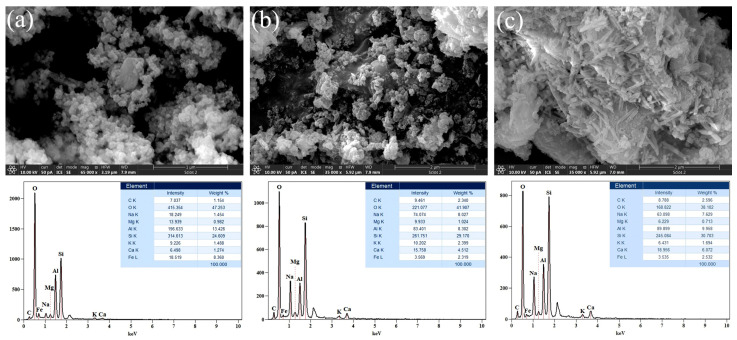
FESEM micrographs and corresponding EDS spectra of alkali-activated, AWAFA, materials: (**a**) 4 M, (**b**) 6 M, and (**c**) 12 M.

FESEM images of alkali-activated, AWAFA, materials and corresponding EDS spectra are presented in Figure 4. It can be observed that with increasing molarity, the gel matrix becomes more compatible and denser. Furthermore, it is evident that the morphologies of AWAFA_4_ (Figure 4a) and AWAFA_6_ (Figure 4b) are similar. In particular, a small-particle structure was found for the 4 M sample, while agglomerations of different sizes can be observed for the 6 M sample. The morphology of the 12 M (Figure 4c) sample differs significantly from the 4 M and 6 M samples. The appearance of the rod-like structure was most likely affected by the increase in the AA concentration.

**Figure 5:**
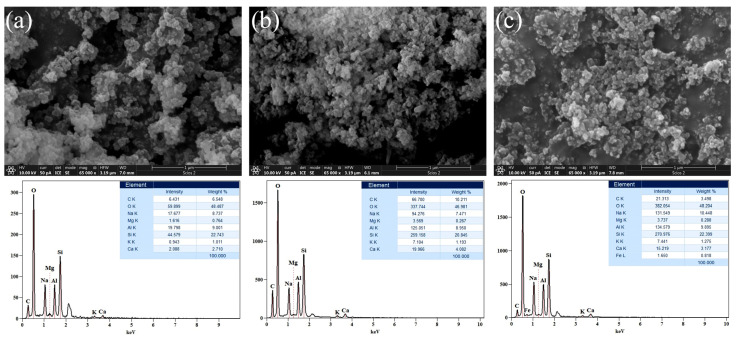
FESEM micrographs and corresponding EDS spectra of alkali-activated, AWAMK, materials: (**a**) 4 M, (**b**) 6 M, and (**c**) 12 M.

Figure 5 shows FESEM micrographs of alkali-activated, AWAMK, materials and corresponding EDS spectra. As can be seen from the obtained images, the morphology of the analyzed samples, for different alkaline activator concentrations, is relatively uniform. Unevenly distributed nanometer-sized particles and their agglomerations of various sizes and shapes are found for all three samples.

**Figure 6:**
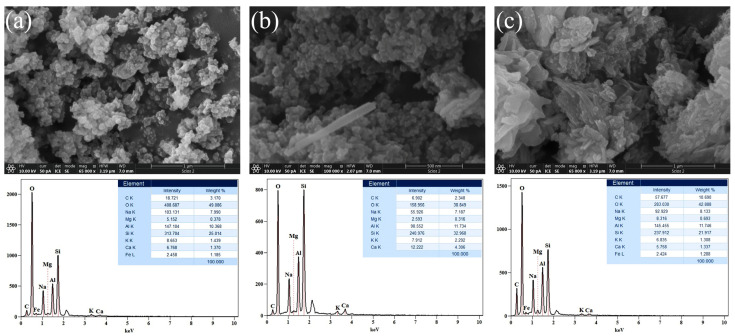
FESEM micrographs and corresponding EDS spectra of the alkali-activated, AWAFAMK, materials: (**a**) 4 M, (**b**) 6 M, and (**c**) 12 M.

Figure 6 shows FESEM micrographs and corresponding EDS spectra of the AWAFAMK samples. Regarding the three-component system that contains an equal amount of the precursors FA and MK (45% + 45%) and 10% of WA, Figure 6 illustrates how the morphology of the samples changes depending on the molarity of the alkaline activator. It is evident from the Figure 6a that the sample is composed of particles with different dimensions and shapes, as well as their agglomerations. The sample depicted in Figure 6b exhibits a rod-like structure that is sporadically visible, aside from small particles on the agglomerate’s surface. As the molarity increases (Figure 6c), the flats and rods present in the gel matrix of samples cluster in a nonuniform manner.

The major elements in the structure of the investigated AAMs are O, Si, Al, Na, Ca, and C, while Mg and K are present in small percentages. The content of Ca, which is present in a large percentage in WA, varies across all the AAMs: for AAM_4_, it ranges from 1.27 to 2.71%; for AAM_6_, from 4.08 to 4.51%, and for AAM_12_, from 1.34 to 6.07 by weight %. The most uniform values of weight % of Ca are observed with all AAM_6_ materials. Sodium is present in a small percentage in FA as a precursor, while it is not detected in WA and MK. Therefore, Na is primarily introduced into the system through the alkali activation process. The weight % of Na for most samples falls within the range of 7–8, regardless of the concentration of the alkali activator. Part of the Na participates in the ion exchange with Cd ions.

#### 2.1.4. BET Analysis

Adsorption involves the removal of Cd ions by solid surfaces of synthesized materials. In order to form fine particles and achieve a sufficiently mesoporous surface of alkaline activated materials, different solid precursors were used. The specific surface area of samples, SBET, calculated by the BET equation is presented in Table 1.

The specific surface area of the AWAFA_6_ and AWAFAMK_6_ samples was 62 m^2^/g and 60 m^2^/g, respectively. The pore radius lies between 1.6 and 32 nm, indicating that the investigated samples are micro- and mesoporous in accordance with IUPAC classification (micropores ≤2 nm, mesopores 2–50 nm, and macropores ≥ 50 nm). The microstructure of the AAMs (Figure 4, Figure 5 and Figure 6) is on the nanometric scale (50–100 nm) and consists of several pores within a highly porous amorphous matrix. Part of the Cd ions are encapsulated in the Si-O or Al-O tetrahedral network, as well as in the meso and micropores of the alkali-activated materials. These pores likely provide space for ion incorporation, substitution, and balancing. Adhesion forces are created between the Cd ions and mesopores which is one of the reasons for the adsorption process [40].

### 2.2. Adsorption of Cadmium Ions

The batch adsorption process experiments were conducted at room temperature to investigate the effect of the experimental conditions in order to conclude which condition achieves the highest amount of cadmium removal; parameters such as the contact time and initial concentrations of Cd(II) were taken; pH and the mass of adsorbents were also tested. The isotherm and kinetics were also characterized in this study.

#### 2.2.1. Effect of Contact Time

**Figure 7:**
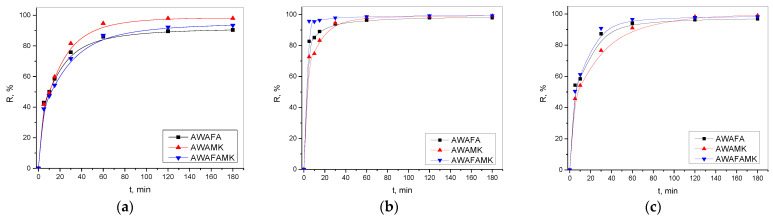
Influence of contact time and effect of concentration of NaOH in alkali activation solution (**a**) 4 M, (**b**) 6 M, and (**c**) 12 M on adsorption capacity using different AAMs.

The effect and influence of contact time (range was from 5 to 180 min) on the adsorption process was analyzed to determine the adsorption equilibrium time. In these experiments, the mass of AAMs was 0.02 g and the initial cadmium concentration was 20.0 mg/dm^3^. It can be concluded, according to the results shown in Figure 7, that the process of adsorption is fast, and the equilibrium was achieved after 60 min. For further investigation of the effects of other process parameters, a time of 120 min was chosen.

According to Onutai et al. (2018), with increasing contact time, the maximum value of efficient removal of Cd(II) ions onto fly-ash-based geopolymer particles was 76.33% in 120 min [73]. In our study, the value of removal efficiency depended on the type of AAM and the concentration of NaOH in the alkali activation solution. The highest removal efficiencies, around 98%, were achieved for all three AAMs at a concentration of 6 M NaOH in 120 min. Likewise, the removal efficiency of cadmium ions using materials prepared with a NaOH concentration of 4 M and 12 M was high and in the range of 85–95%. Based on the results presented in Figure 7, it can be seen that for all three types of mixture, materials prepared with NaOH concentrations of 4 M and 12 M showed a very similar trend (slower adsorption and lower adsorption efficiency values), whilst the behavior of materials with a NaOH concentration of 6 M is different (fast adsorption and highest adsorption efficiency values).

#### 2.2.2. Effect of Initial Cadmium Concentration

**Figure 8:**
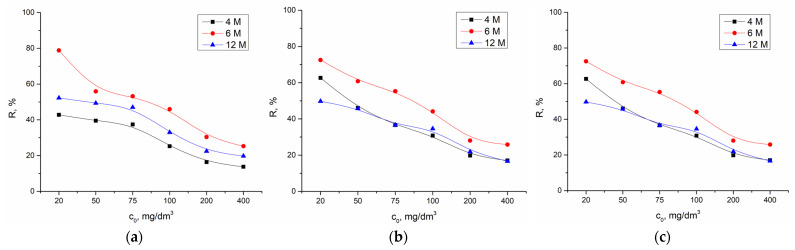
Influence of the initial concentration of cadmium and effect of the concentration of NaOH in the alkali activation solution on adsorption efficiency using (**a**) AWAFA, (**b**) AWAMK, and (**c**) AWAFAMK.

**Figure 9:**
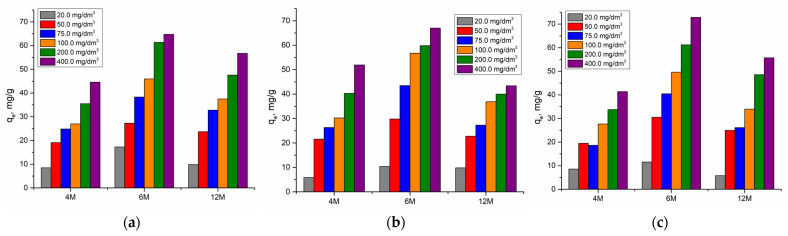
Influence of the initial concentration of cadmium and effect of the concentration of NaOH in the alkali activation solution on adsorption capacity using (**a**) AWAFA, (**b**) AWAMK, and (**c**) AWAFAMK.

Figure 8 and Figure 9 show the effects of the initial cadmium ions concentrations and the effect of the concentration of NaOH in the alkali activation solution on adsorption efficiency and capacity. The effects of varying the concentration of Cd(II) on the removal efficiency were tested in the concentration range from 20.0 mg/dm^3^ to 100.0 mg/dm^3^, with a mass of 0.02 g AAMs at pH 6.5, room temperature, and a contact time of 120 min.

According to expectation, increasing the initial concentration decreases the removal efficiency (Figure 8). Since the amount of adsorbent is constant, with an increase in initial concentration of cadmium ions, saturation of the active sites on AAMs occurs. The highest value for all materials was reached at the initial concentration of 20.0 mg/dm^3^.

As can be seen in Figure 9, the adsorption capacity increases with increasing initial cadmium concentration. At the same time, the influence of the concentration of NaOH in the alkaline activation solution was noticeable for all precursor materials, waste materials such as FA and WA, and natural material like MK. The highest values were achieved at a concentration of 6 M.

#### 2.2.3. Effect of pH Value

**Figure 10:**
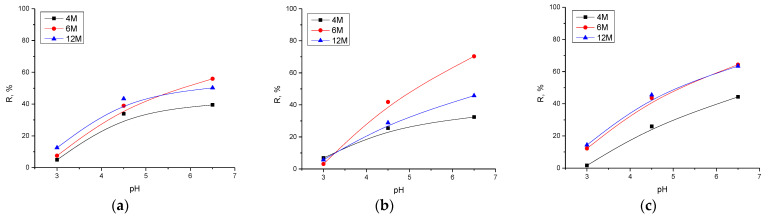
Influence of pH value and effect of the concentration of NaOH in the alkali activation solution on adsorption efficiency using (**a**) AWAFA, (**b**) AWAMK, and (**c**) AWAFAMK.

**Figure 11:**
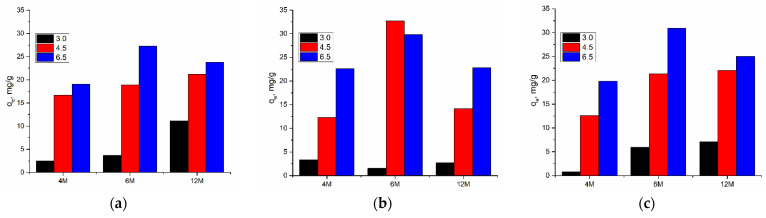
Influence of pH value and effect of the concentration of NaOH in the alkali activation solution on adsorption capacity using (**a**) AWAFA, (**b**) AWAMK, and (**c**) AWAFAMK.

The influence of pH on the adsorption capacity of the AAMs was examined within the pH range from 3.0 to 6.5, with the mass of adsorbents of 0.02 g, and the concentration of cadmium ions of 50.0 mg/dm^3^. The pH value was adjusted with a small amount of 0.1 M and 0.5 M HCl. The dependency of the adsorption capacity on the pH value is displayed in Figure 10 and Figure 11.

An increase in pH values positively effects the values of adsorption capacity. Again, the highest values of capacity were achieved using materials prepared with the 6 M NaOH concentration. A weakly acidic and close to neutral environment favors the binding of cadmium because the concentration of H^+^ ions competing for the same active binding sites is reduced. A further increase in the pH value was not investigated due to the possibility of cadmium hydroxide precipitation. At values above 7, the appearance of CdOH^+^ ions and the reduction of Cd(II) occurs, which could also affect the adsorption process.

### 2.3. Adsorption Ishoterms

**Figure 12:**
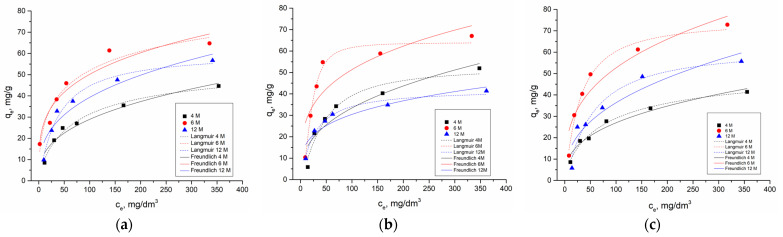
Nonlinear fit of the Langmuir and Freundlich models of (**a**) AWAFA, (**b**) AWAMK, and (**c**) AWAFAMK in dependence of the concentration of NaOH in the alkali activation solution.

Figure 12 shows the nonlinear fit of the Langmuir and Freundlich models of all of the AAMs depending on the concentration of NaOH in the alkali activation solution. Parameter values for both models are presented in Table 2. The high value of the correlation coefficient indicates that the fit of the experimental results for all of the AAMs is better with the Langmuir adsorption isotherm.

### 2.4. Adsorption Kinetics

Nonlinear models of pseudo-first and pseudo-second order were used to fit the experimental data. Calculated values of kinetic parameters for pseudo-first and pseudo-second order are presented in Table 3 for the investigated AAMs.

**Figure 13:**
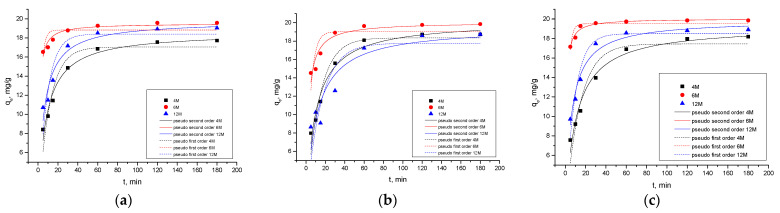
Nonlinear fit of the pseudo-first and pseudo-second order model of (**a**) AWAFA, (**b**) AWAFA, and (**c**) AWAFA in dependence of the concentration of NaOH in the alkali activation solution.

According to the results in Table 3 and Figure 13, it can be concluded that the correlation coefficient values closer to 1 indicate that cadmium ions adsorption kinetics can be represented by the pseudo-second order and presumed chemisorption mechanism. Rasaki et al. (2019) concluded that fly-ash/metakaolin-based geopolymers often show a remarkable adsorption capacity towards divalent metal ions with a pseudo-second-order adsorption process. This suggests that the heterogeneous surface of the geopolymers is favorable for the removal of the metal ions [74].

According to this research, the alkali activation of precursors such as metakaolin, wood ash, and fly ash could be efficiently used for the removal of cadmium ions from wastewater. Also, the experimental conditions of the alkali activation process, such as the concentration of alkali solution and percent ratio of precursors have an important influence on the adsorption properties. It can be observed that, in most cases, the alkali-activated materials that were synthesized by 6 M NaOH in an alkali solution have the highest value for removal efficiency of cadmium ions. The concentration of the NaOH solution is an important factor in controlling the properties of AAMs. Higher concentrations of NaOH create a more alkaline environment, enhancing the dissolution of reactive aluminosilicates in precursors. This results in a higher degree of polymerization reaction and the formation of aluminosilicate gels. Moreover, increasing the concentration of NaOH leads to the dissolution of Na_2_SiO_3_ solutions, introducing additional silicate species and sodium into the system. Some researchers have reported that the structural stability of geopolymers increased with the addition of silicates, due to the formation of long-chain silicate oligomers and Al-O-Si complexes. However, a very high silicate concentration promotes the formation of Si-rich gels with a high percentage of bridge bonds, resulting in a more amorphous geopolymer, Nevertheless, excess silicate could hinder water evaporation, impeding geopolymer structure formation [75]. In our study, we found that the best adsorption values were obtained with AAM_6_ among the tested materials. The goal of our research was to optimize the process, specifically to determine the most suitable alkaline activator for a given combination of solid precursors, to achieve the best adsorption characteristics in the presence of cadmium in an aqueous solution. However, the use of 12 M NaOH as an alkaline activator poses challenges due to its high pH, hindering the geopolymerization reaction and complicating the washing process. The appearance of new crystalline phases in the three-component system further adversely affects the application of AAM_12_ as an adsorbent.

According to Borah et al. (2018), burmese grape leaf extract (BGLE) was used as a low-cost adsorbent for cadmium (Cd(II)) removal from metal solutions and a natural water sample. The experiments showed the effective adsorption of these ions with a maximum adsorption capacity of 44.72 mg/g [76]. Furthermore, Pehlivan et al. (2008) used sugar beet pulp as a low-cost material for the adsorption of Cd(II) and Pb(II), and the maximum metal sorption capacities were 46.1 mg/g for Cd(II) and 43.5 mg/g for Pb(II) [77]. Srivastava et al. (2006) investigated the adsorption of cadmium (Cd(II)) and nickel (Ni(II)) ions onto bagasse fly ash (BFA) from single-component and binary systems [78]. According to Srivastava et al. (2006)’s investigations, it was obtained that when the initial ion concentration increases from 10 to 100 mg/l, the loading capacity of BFA increases from 0.89 to 5.18 mg/g for Cd(II) and from 0.95 to 5.78 mg/g for Ni(II) [78]. The alkaline treatment of algae waste biomass for increasing the biosorption capacity was investigated by Dumitru and Laura Bulgariu (2016), and the acquired results show higher biosorption capacity of 41.88 mg/g compared to 33.71 mg/g for untreated algae waste biomass [79]. Zhao et al. (2020) investigated the sodium-hydroxide-modified fly ash (SHM-FA), and under optimal conditions the adsorption capacity of Cd(II) was 31.79 mg/g [80].

The development of low-cost sorbents based on waste and natural materials or biomass is the subject of many current scientific research studies. In this way, it is possible to remove pollutants from the water and also achieve the reuse of waste materials and reduction in their disposal in the environment. The results of adsorption capacity obtained in this study range from 8.48 to 72.84 mg/g for Cd(II), and show great potential for the application of alkali-activated materials based on a mixture of fly ash and metakaolin with the addition of wood ash for the removal of cadmium.

## 3. Conclusions

XRD analysis indicates the presence of quartz and albite phases in AAMs originating from the FA and MK precursors, while calcite and portlandite are derived from WA. Upon the activation of all samples, new crystalline phases only emerge in the AWAFAMK_12_ sample. A characteristic “halo” in the XRD patterns, centered around 20°–40° 2*θ*, representing a new amorphous gel, is observed in FA and all AAMs containing FA. The DRIFT spectra of all raw materials show a band displacement at 1047 cm^−1^ after alkaline activation to a wavenumber in the range between 1021 cm^−1^ and 1036 cm^−1^. This displacement can be attributed to the newly designed asymmetric stretching vibration of Si-O-Si or Si-O-Al, characteristics of the polymer structure of AAMs.

The FESEM analysis of alkali-activated materials revealed that an increase in the concentration of NaOH in the activator solution had an impact on the rod-like structure’s appearance in the AWAFA samples. The morphology of the AWAMK samples, as a function of the concentration of the alkaline activator, is quite uniform. The FESEM micrographs of the three-component system show the contribution of each of the three components when subjected to the influence of an alkaline activator of different molarity. As the molarity of alkali activator increases, the flats and rods present in the sample cluster in a non-uniform manner.

The static adsorption tests showed that the pseudo-second-order kinetic model and Langmuir adsorption isotherm model gave the best fit, indicating that the monolayer chemisorption interactions may play a dominant role in the adsorption of cadmium by alkali-activated materials based on fly ash and metakaolin with the addition of wood ash. Adsorbent behavior was examined as a function of the initial concentration of cadmium ions, contact time, pH value, and mass. At the lower concentration of cadmium ions, the efficiency of removal was higher in regards to the higher concentration of cadmium ions in all investigated alkali-activated materials. According to the acquired results, equilibrium was reached after 120 min and the efficiency removal was above 90%. The Langmuir adsorption isotherm and pseudo-second-order kinetic model provided a better fit for the experimental results, confirming the chemisorption of Cd ions onto alkali-activated materials. During the polymerization of various mixes of solid precursors, new cation exchange sites were created. Sodium ions were available for ion exchange with cadmium ions. Consequently, more than one mechanism was responsible for the Cd(II) adsorption process by the investigated alkali-activated materials.

It is known that fly ash especially has a negative impact on the environment due to the increased leaching of pollutants. Also, the alkaline activation of wood ash, fly ash, and metakaolin affected the improvement of the adsorption properties of the obtained materials and the stabilization of the ashes. In this way, the leaching of polluting substances is reduced, and the used adsorbents can be used later as additives for the production of building materials.

## 4. Materials and Methods

### 4.1. Synthesis of Alkali-Activated Materials

To produce alkali-activated materials (AAMs), the solid precursors fly ash (FA), wood ash (WA), and metakaolin (MK) were employed. Metakaolin was obtained after thermal treatment of kaolin (Rudovci, Serbia) in a laboratory furnace until 750 °C (heating rate 10°/min, and soaking time 1 h at this elevated temperature), and after that spontaneously cooling at room temperature [81,82]. Fly ash was obtained as a byproduct of lignite combustion at the thermal power plant “Nikola Tesla” in Obrenovac, Serbia, while the obtained wood ash resulted from burning oak trees in individual fireplaces. The synthesis of AAMs involved mixing different masses of these solid precursors and an alkali activator (AA) (see Table 4). The samples were marked as follows: AWAFA_4_, AWAFA_6_, AWAFA_12_; AWAMK_4_, AWAMK_6_, AWAMK_12_; AWAFAMK_4_, AWAFAMK_6_, and AWAFAMK_12_ (where A—abbreviation of AAM, WA—wood ash, MK—metakaolin, FA—fly ash, and subscript number represents the concentration of solution of NaOH in alkali activator solution).

Alkali activator (AA) comprises two compounds: a sodium hydroxide solution (Sigma-Aldrich, St. Louis, MO, USA) and a sodium silicate solution (Interhem company, Belgrade, Serbia). Sodium hydroxide solutions were prepared in three concentrations (4 M, 6 M, and 12 M). The sodium silicate molar modulus (SiO_2_/Na_2_O) was 3.1. Sodium hydroxide and sodium silicate solutions were stirred on a magnetic mixer for three hours and prepared one day before use. The volume ratio of sodium hydroxide solution to sodium silicate solution was 1.5. Due to the different suspension workability functions, the mass ratio of the solid to liquid phase was approximately 0.8–1.0.

The determined volume of AA and mass of precursors were briefly mixed for less than 5 min, poured into molds, covered, and left at room temperature (23 ± 1) for twenty-four hours. Subsequently, the samples were placed in a laboratory oven at sixty degrees for an additional two days in molds with covers. After drying in the oven, the obtained materials were left for weeks at room temperature under monitored conditions, temperature, and relative humidity. Finally, the specimens were demolded and related experiments were performed. The alkali-activated samples were crushed, sieved, and washed with distilled water while monitoring the pH of the washed solution until it reached 7. After that, the AAMs were prepared for characterization and applied for the adsorption process.

### 4.2. Technics for Analysis of Raw and Alkali-Activated Materials

#### 4.2.1. XRF Analysis

Table 5 presents the chemical composition of the raw samples WA, FA, and MK, determined through X-ray fluorescence (XRF) analysis. The XRF analysis was performed with a wavelength dispersion (WD XRF) spectroscope ARL Perform X manufactured by Thermo Scientific (Houston, TX, USA) with a power of 2500 W, 5 GN Rh X-ray tube, 4 crystals (AX03, PET, LiF200 and LiF220), two detectors (proportional and scintillation), and computer program UniQuant. The samples were quartered, dried at 105 °C, and calcined at 950 °C. For measurement purposes, a fused pellet was prepared, where 0.7640 g of the sample and 7.64 g of the flux (50% lithium tetraborate versus 50% lithium metaborate) were melted at 1100 °C.

The XRF results indicate that CaO is the predominant compound in WA (more than 30%), while FA and MK are primarily composed of Al_2_O_3_ and SiO_2_. The loss on ignition (LoI), representing the amount of unburned carbon, is highest in WA.

#### 4.2.2. DRIFT Spectroscopy

The functional groups of all samples were determined by diffuse reflectance infrared Fourier transform (DRIFT) spectroscopy. The DRIFT spectra were obtained using the Perkin-Elmer FTIR spectrometer Spectrum Two [83]. The spectra of investigated samples were scanned at 4 cm^−1^ resolution and collected in the mid-IR region from 4000 to 400 cm^−1^.

#### 4.2.3. XRD Analysis

The mineralogy composition of raw materials and AAMs was determined by the XRD method using the Ultima IV Rigaku diffractometer (Rigaku, Tokyo, Japan). A range of 5–80° 2*θ* with a scanning step size of 0.02° and a scan rate of 5°/min in a continuous scan mode was used. The PDXL2 software 2.8.4.0 (Rigaku, Tokyo, Japan) [84] was used to evaluate the phase composition and identification mineralogical compositions of all samples. All obtained patterns were compared using the International Crystallographical Database (ICDD) [85].

#### 4.2.4. FESEM-EDS Spectroscopy

Field emission scanning electron microscope, equipped with energy dispersive X-ray spectroscopy (FESEM-EDS, FEI Scios2, Dual Beam system, Thermo Fisher Scientific, Houston, TX, USA) was employed for morphological studies, as well as for elemental analysis of the samples. Before imaging, the powder samples were attached to a sample holder using double-sided copper tape and then sputter-coated with gold to make them conductive. The micrographs were taken at an acceleration voltage of 10 kV and a chamber pressure of approximately 9 × 10^−5^ Pa.

#### 4.2.5. BET Analysis

The specific surface area and pore size distribution (PSD) were analyzed using Surfer (Thermo Fisher Scientific, Houston, TX, USA). Prior to analysis, the investigated samples underwent degassing under vacuum. The pore size distribution (PSD) was estimated using the BJH (Barrett–Joyner–Halenda) method [86], applied to the desorption branch of isotherms. Additionally, the mesopore surface area and micropore volume were determined using the t-plot method [87].

### 4.3. Adsorption of Cadmium

Examinations of the effect of some parameters on the efficacy of the AAMs for cadmium adsorption were derived by adsorption experiments at room temperature in a batch process. A stock solution of the Cd(II) ion was made by dissolving the salt of cadmium in deionized water. The pH value of the solutions was regulated with HCl solution. After adsorption, the solution was centrifuged and analyzed for cadmium concentration by flame atomic absorption spectrophotometry (FAAS PyeUnicam) at 228.8 nm. The contact time, initial concentrations, mass of adsorbent, and pH of the sample solutions were analyzed to study their effect on the removal efficiency of Cd(II) by the AAMs.

The removal efficiency [88], *R* (*%*), of the AAMs was calculated with Equation (1):

(1) $$R=\frac{\left(c_{0}-c_{e}\right)}{c_{o}}\cdot 100$$

where the *c*_0_ and *c_e_* (mg/L) are the initial and the equilibrium concentration of Cd(II) in the solution, respectively.

The adsorption capacity [88], *q_e_* (mg/g), was calculated with Equation (2):

(2) $$q_{e}=\frac{\left(c_{0}-c_{e}\right)V}{m}$$

where *V* represents the volume of the solution and *m* is the mass of the adsorbent.

The Langmuir and Freundlich isotherm models were used to fit experimental data in order to find the mechanism of the adsorption process. The Langmuir model was initially developed assuming monolayer adsorption on a surface of the adsorbent with a finite number of adsorption sites. The nonlinear form of the Langmuir equation [88] is presented in Equation (3):

(3) $$q_{e}=\frac{q_{m}K_{L}c_{e}}{1+K_{L}c_{e}}$$

The Freundlich model assumes that adsorption takes place on heterogeneous surfaces of the adsorbent. The nonlinear form of the Freundlich equation [88] is represented by Equation (4):

(4) $$q_{e}=K_{f}+{c_{e}}^{\frac{1}{n}}$$

The pseudo-first-order equation describes adsorption in solid–liquid systems based on the sorption capacity of solids.

The pseudo-first-order nonlinear reaction model [88] is expressed as the following Equation (5):

(5) $$q_{t}=q_{e}\left(1-{exp}^{{-k}_{1}t}\right)$$

The pseudo-second model is based on the assumption of chemisorption of the adsorbate on the adsorbent. The nonlinear model is expressed as (6) [88]:

(6) $$q_{t}=\frac{k_{2}q_{e}^{2}t}{1+k_{2}q_{e}t}$$

## Figures and Tables

**Table 1:** Porous properties of AWAFA_6_ and AWAFAMK_6_ samples.

Sample	S_BET_ (m^2^/g)	S_meso_ (m^2^/g)	S_mic_ (m^2^/g)	V_mic_ (cm^3^/g)	r_med_ (nm)
AWAFA_6_	62	56	6	0.0041	13.37
AWAFAMK_6_	60	55	5	0.0029	10.52

**Table 2:** Adsorption isotherm parameter for the Langmuir and Freundlich models for AAMs.

**The Langmuir Model**	**Adsorbent**								
	**AWAFA**			**AWAMK**			**AWAFAMK**		
	**4 M**	**6 M**	**12 M**	**4 M**	**6 M**	**12 M**	**4 M**	**6 M**	**12 M**
K_L_, dm^3^/mg	3.02 × 10^−2^	2.07 × 10^−2^	1.02 × 10^−1^	7.57 × 10^−3^	1.77 × 10^−3^	2.79 × 10^−2^	3.11 × 10^−2^	1.08 × 10^−2^	2.09 × 10^−2^
*q_m_*, mg/g	56.42	60.59	111.0	52.77	63.95	42.02	54.44	62.13	75.19
R^2^	0.9764	0.9719	0.9178	0.9332	0.9734	0.9682	0.9835	0.9149	0.9784
**The Freundlich model**	**Adsorbent**								
	**AWAFA**			**AWAMK**			**AWAFAMK**		
	**4 M**	**6 M**	**12 M**	**4 M**	**6 M**	**12 M**	**4 M**	**6 M**	**12 M**
*K_f_*	5.342	8.196	14.73	5.617	14.16	8.648	4.964	5.734	11.64
n	2.726	2.938	3.761	2.582	3.578	3.661	2.721	2.494	3.052
R^2^	0.9484	0.8978	0.9149	0.8604	0.6980	0.8579	0.9546	0.8559	0.8825

**Table 3:** Adsorption kinetics parameter of pseudo-first and pseudo-second order for AAMs.

**Pseudo-First Order**	**Adsorbent**								
	**AWAFA**			**AWAMK**			**AWAFAMK**		
	**4 M**	**6 M**	**12 M**	**4 M**	**6 M**	**12 M**	**4 M**	**6 M**	**12 M**
k_1_, 1/min	0.089	0.384	0.113	0.075	0.219	0.065	0.071	0.399	0.109
*q_e_*, mg/g	17.05	18.82	18.40	18.36	19.02	17.77	17.44	19.51	18.49
R^2^	0.89039	0.43702	0.82105	0.93408	0.57442	0.70992	0.90351	0.72182	0.92165
**Pseudo-second order**	**Adsorbent**								
	**AWAFA**			**AWAMK**			**AWAFAMK**		
	**4 M**	**6 M**	**12 M**	**4 M**	**6 M**	**12 M**	**4 M**	**6 M**	**12 M**
k_2_, g/mg min	6.89 × 10^−3^	4.74 × 10^−2^	9.05 × 10^−3^	5.05 × 10^−3^	2.06 × 10^−2^	4.72 × 10^−3^	5.02 × 10^−3^	5.82 × 10^−2^	8.51 × 10^−3^
*q_e_*, mg/g	18.59	19.51	19.76	20.21	20.06	19.56	19.28	20.03	19.90
R^2^	0.99656	0.97341	0.98208	0.98732	0.96626	0.94157	0.99064	0.97382	0.98925

**Table 4:** The mass of solid precursors and volume of alkali activator for the synthesis of AAMs.

AAMs	WA (g)	FA (g)	MK (g)	AA (cm^3^)
AWAFA_4_	1.0	9.0		10
AWAFA_6_	1.0	9.0		10
AWAFA_12_	1.0	9.0		10
AWAMK_4_	1.0		9.0	9.0
AWAMK_6_	1.0		9.0	9.0
AWAMK_12_	1.0		9.0	9.0
AWAFAMK_4_	1.0	4.5	4.5	10
AWAFAMK_6_	1.0	4.5	4.5	10
AWAFAMK_12_	1.0	4.5	4.5	10

**Table 5:** Chemical composition of WA, FA, and MK.

Chem.	Na_2_O	MgO	Al_2_O_3_	SiO_2_	P_2_O_5_	SO_3_	K_2_O	CaO	TiO_2_	MnO	Fe_2_O_3_	ZnO	As_2_O_3_	BaO	L.O.I. *
WA (wt.%)	0.51	4.25	4.06	4.07	1.92	1.18	11.16	38.76	0.11	1.43	0.72	0.19	0.14	0.26	31.06
FA (wt.%)	0.32	2.07	27.36	55.9	0.07	0.18	1.49	3.69	0.67	0.07	5.93	0.01	0.15	0.08	1.79
MK (wt.%)	0.17	0.53	31.23	60.85	0.03	/	2.29	0.40	0.65	0.01	1.92	0.01	0.08	0.05	1.61

* 950 °C.

## Data Availability

All data and materials are available on request from the corresponding author. The data are not publicly available due to ongoing researches using a part of the data.
